# Progress in metathesis chemistry II

**DOI:** 10.3762/bjoc.11.179

**Published:** 2015-09-15

**Authors:** Karol Grela

**Affiliations:** 1 Biological and Chemical Research Centre, Faculty of Chemistry, University of Warsaw, Żwirki i Wigury 101, 02-089 Warsaw, Poland; 2 Institute of Organic Chemistry, Polish Academy of Sciences, Kasprzaka 44/52, 01-224 Warsaw, Poland

**Keywords:** olefin metathesis


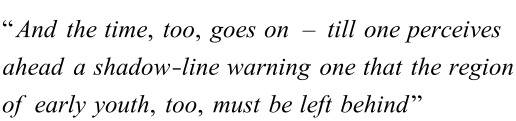
 

Joseph Conrad – The Shadow-Line

Five years have passed since the first publication of the Thematic Series on Olefin Metathesis in the *Beilstein Journal of Organic Chemistry* [1]. During these years the research continued to progress at full speed. Astute readers of this Thematic Series, as well as readers of the recent books devoted to olefin metathesis [2–3], can easily see that a great number of studies in this field have advanced from the basic research phase to the commercial application stage. While new, active and more selective catalysts that solve some longstanding limitations are still being developed, a growing number of projects deal only with applications using olefin metathesis as one of many stock transformations.

However, this does not imply that the research phase is over, that all problems have been solved, that the technology is widely recognized and used, and that catalyst manufacturers have become millionaires. It only means that olefin metathesis is now a full-grown technology, which has already crossed the shadow line – the term coined by the Polish–British novelist Józef Teodor Konrad Korzeniowski (Joseph Conrad) to indicate the point where maturity is gained. This maturity will bring new promise, new expectations, and new challenges. During the application of this technology, new problems on various levels will surely emerge. I am therefore fully convinced that in the forthcoming years scientists dealing with olefin metathesis will have many opportunities for exciting research and for hard work, too.

On a personal level, it was for me a great pleasure to serve as the editor of this Thematic Series. I am very thankful to all authors for their first-class contributions. At the same time, I would like to thank the colleagues at the Beilstein-Institut for their professional support and patience.

Karol Grela

Warsaw, August 2015
